# Supplementary material for: Multiple mechanisms contribute to fluorometry signals from the voltage-gated proton channel
Source: Commun Biol. 2022 Oct 26;5:1131. doi: 10.1038/s42003-022-04065-6 (PMC9606259; doi:10.1038/s42003-022-04065-6)
Supplement: Supplementary file 2 — Supplementary Material [file 42003_2022_4065_MOESM2_ESM.pdf]

a

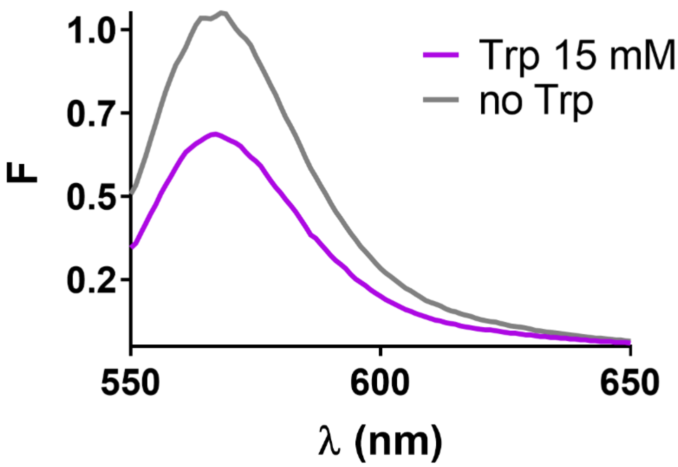

b

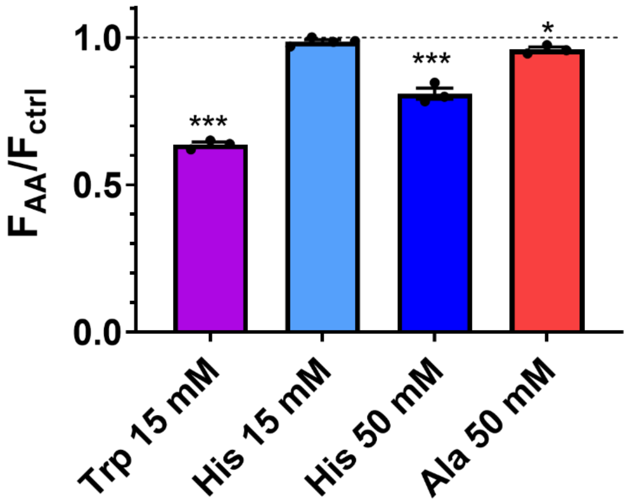

Supplementary Figure 1. Quenching of TAMRA-MTS by amino acids.

(A) Quenching of TAMRA-MTS fluorescence by tryptophan (15 mM) in aqueous solution. (B) Average fluorescence of TAMRA-MTS solution in the presence of amino acids (Trp, His, Ala), normalized to TAMRA-MTS fluorescence in the absence of amino acids. Error bars represent SEM, * indicates significant difference (* $p<0.05$, *** $p<0.001$). Data were compared to 1 using one-way ANOVA ($N \geq 3$).

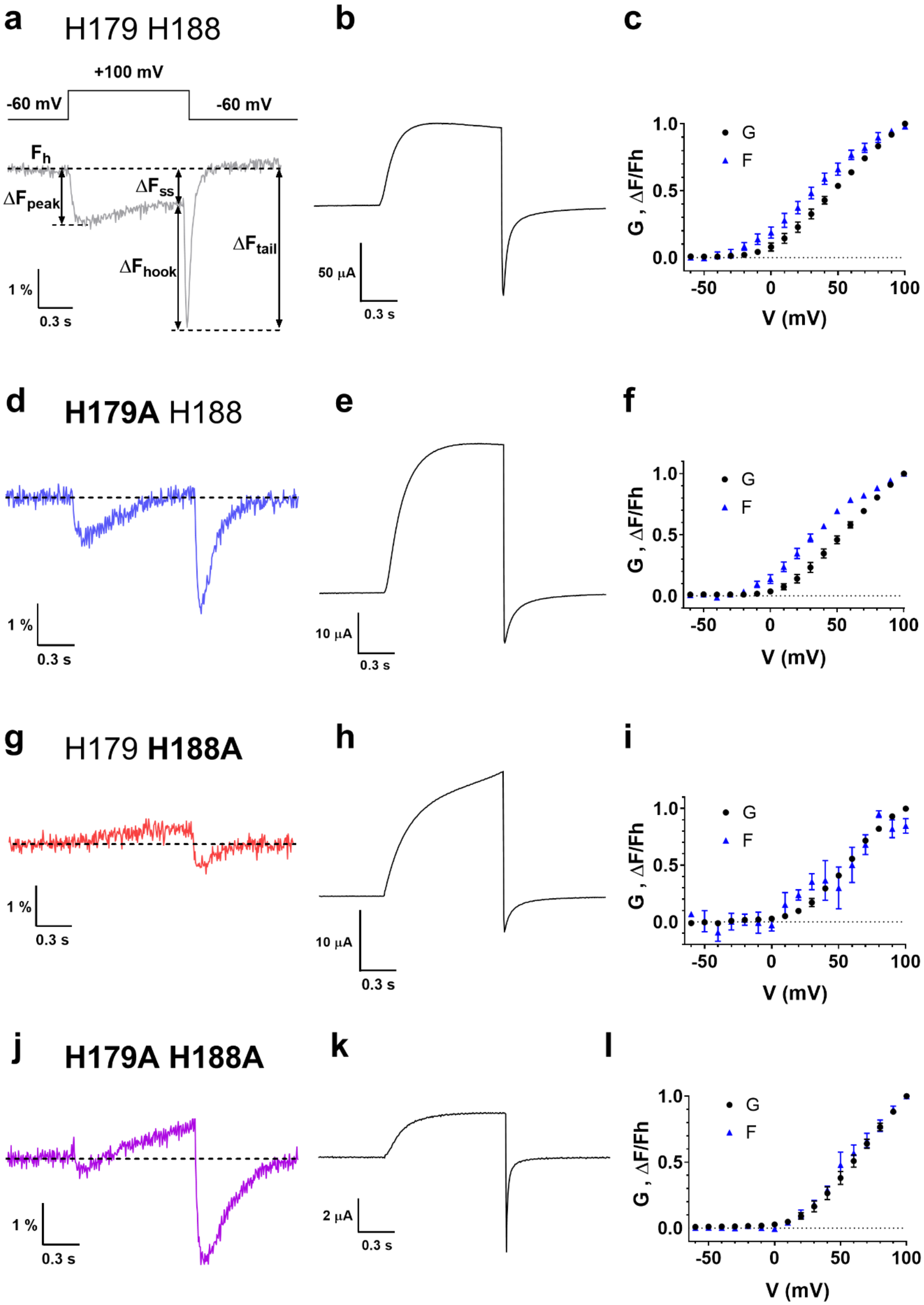
a
H179 H188
+100 mV
-60 mV
-60 mV
Fh
ΔFpeak
ΔFss
ΔFhook
ΔFtail
1 %
0.3 s
b
50 μA
0.3 s
c
G
F
G , ΔF/Fh
V (mV)
d
H179A H188
1 %
0.3 s
e
10 μA
0.3 s
f
G
F
G , ΔF/Fh
V (mV)
g
H179 H188A
1 %
0.3 s
h
10 μA
0.3 s
i
G
F
G , ΔF/Fh
V (mV)
j
H179A H188A
1 %
0.3 s
k
2 μA
0.3 s
l
G
F
G , ΔF/Fh
V (mV)

Supplementary Figure 2. Fluorescence and current traces of CiHv1-E241C and alanine mutants**.**

Representative fluorescence responses (left panels) to a voltage step from -60 mV to +100 mV from oocytes labeled with TAMRA-MTS, expressing CiHv1-E241C (A) and different alanine mutants: H179A (B), H188A (C) and H179A H188A (D) in the E241C background. Middle panels show the proton currents measured simultaneously with the fluorescence traces, while right side panels show the G-V and F-V functions. F_h is the fluorescence at the holding voltage of -60 mV, ΔF represents ΔF_{tail}. Error bars represent SEM (for H179 H188 N=5; for H179A H188 N=3; for H179 H188A N=4; for H179A H188A N=3).

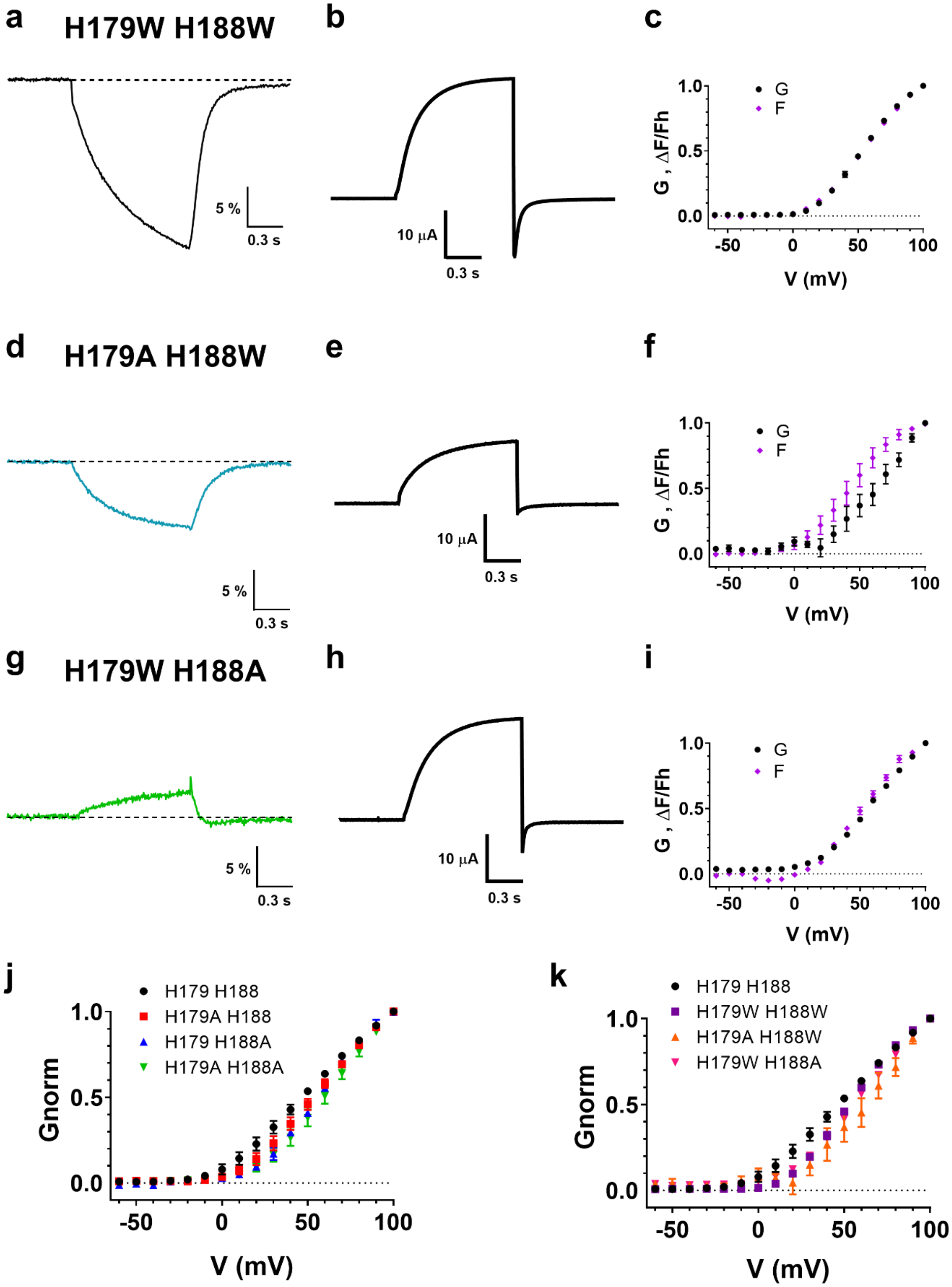

a
H179W H188W
5 %
0.3 s
b
10 μA
0.3 s
c
G
F
G , ΔF/Fh
V (mV)
d
H179A H188W
5 %
0.3 s
e
10 μA
0.3 s
f
G
F
G , ΔF/Fh
V (mV)
g
H179W H188A
5 %
0.3 s
h
10 μA
0.3 s
i
G
F
G , ΔF/Fh
V (mV)
j
H179 H188
H179A H188
H179 H188A
H179A H188A
Gnorm
V (mV)
k
H179 H188
H179W H188W
H179A H188W
H179W H188A
Gnorm
V (mV)

Supplementary Figure 3. Fluorescence and current traces of CiHv1 tryptophan mutants**.**

Representative fluorescence responses (left panels) to a voltage step from -60 mV to +100 mV from oocytes labeled with TAMRA-MTS, expressing CiHv1 E241C with different tryptophan mutants: H179W H188W (A), H179A H188W (B), H179W H188A (C). Middle panels show the proton currents measured simultaneously with the fluorescence traces, while right side panels show the G-V and F-V functions. Conductance and fluorescence points almost completely overlap on the right panel of (A). F-V means ΔF/F – V for all tryptophan constructs. Panels D and E summarize the G-V functions of the various constructs. Error bars represent SEM (for H179W H188W N=4; for H179A H188W N=5; for H179W H188A N=3).

a

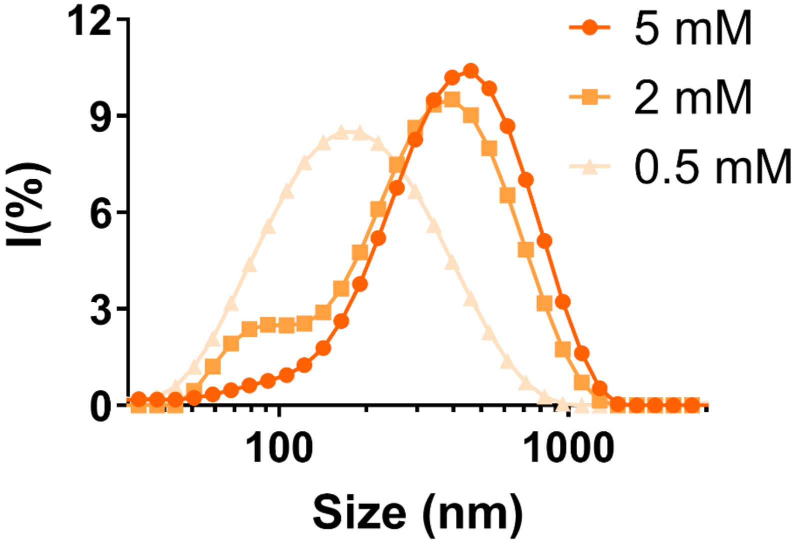

b

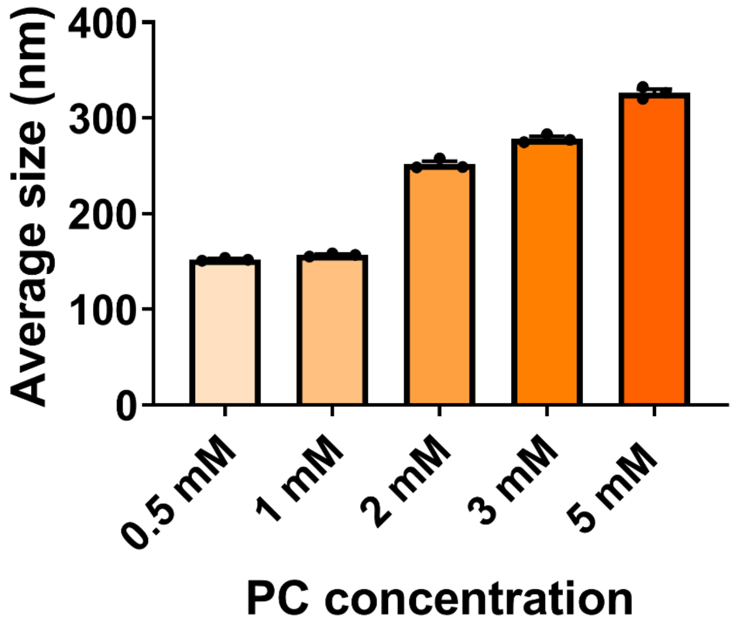

Supplementary Figure 4. PC vesicle size distribution at different PC concentrations

(A) Hydrodynamic size distribution of the PC vesicles at different PC concentrations. (B) Average PC vesicle sizes at different PC concentrations. Error bars represent SEM (N=3).

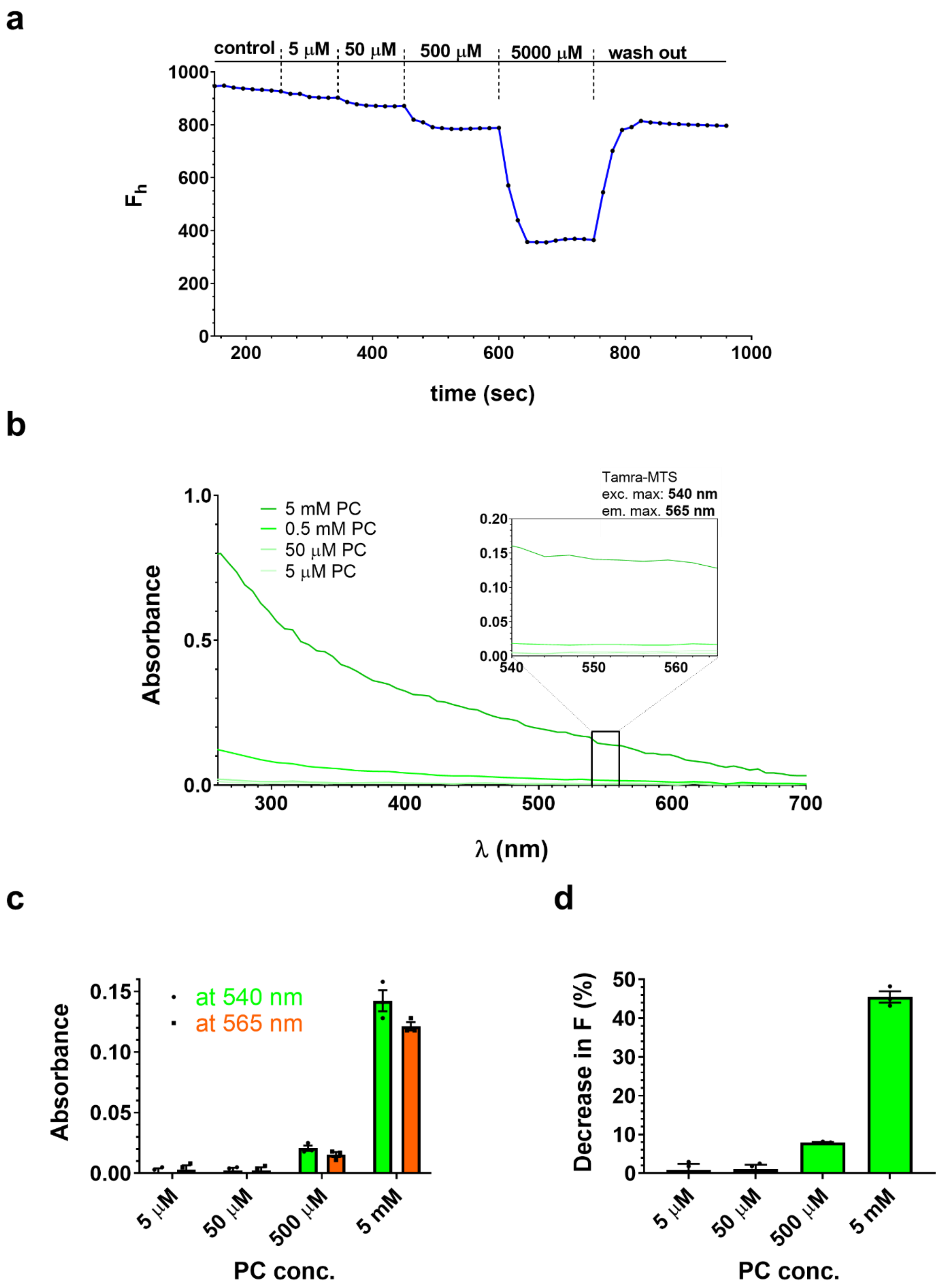

a
control
5 μM
50 μM
500 μM
5000 μM
wash out
F_h
time (sec)
b
5 mM PC
0.5 mM PC
50 μM PC
5 μM PC
Tamra-MTS
exc. max: 540 nm
em. max. 565 nm
Absorbance
λ (nm)
c
at 540 nm
at 565 nm
Absorbance
PC conc.
d
Decrease in F (%)
PC conc.

Supplementary Figure 5. Absorbance of PC

Fluorescence measured at the holding potential (-60mV) as it changes during a representative VCF measurement while the PC concentration changes in the recording extracellular solution. (B) Light absorbance of PC at different concentrations measured by NanoDrop Spectrophotometer. Inset zooms on the range between 540 nm (excitation maximum of TAMRA) and 565 nm (emission maximum of TAMRA). (C) Average light absorbance of PC at 540 nm and 565 nm at different PC concentrations. Error bars represent SEM (N=3). (D) Decrease in TAMRA fluorescence intensity at different PC concentrations, calculated from average absorbance.

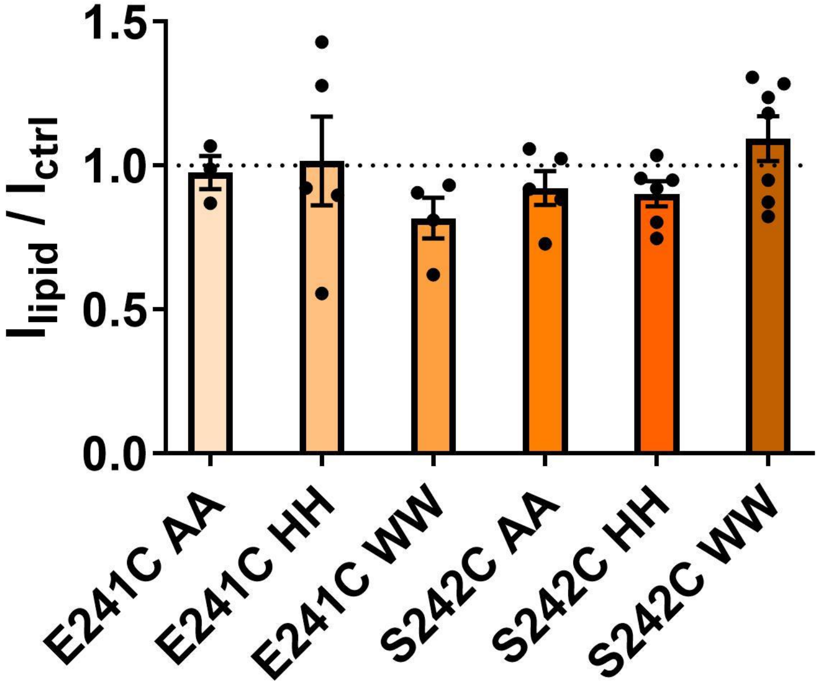

Supplementary Figure 6. Effect of PC (phosphatidylcholine) on proton currents

Average current ratios for different CiHv1 constructs: I_{lipid} / I_{ctrl}, where I_{lipid} represents the measured proton current in the presence of 5mM PC in the extracellular solution, while I_{ctrl} indicates the proton current from the same oocyte without PC molecules. AA abbreviates H179A H188A, HH means H179 H188 and WW stands for H179W H188W. (N ≥ 3)

State	Resting (1)	Transition (1↔2)	Intermediate (2)	Transition (2↔3)	Activated (3)
Free Energy at V = 0 mV (kT)	0	4.5 ± 0.3	2.8 ± 0.4	5.9 ± 0.3	1.6 ± 0.6
Charge (e)	0	0.41 ± 0.08	0.61 ± 0.14	1.18 ± 0.12	2.0 ± 0.2
**H179A H188A**					
F (percent)	100 %	-	85 ± 5 %	-	100.4 ± 0.4 %
**H179A H188**					
F (percent)	100 %	-	85 ± 4 %	-	99.7 ± 0.2 %
**WT H179 H188**					
F (percent)	100 %	-	83 ± 6 %	-	98.4 ± 0.5 %
**H179A H188W**					
F (percent)	100 %	-	96 ± 3 %	-	92 ± 3 %

Supplementary Table 1. Parameters of model calculations for Ci-Hv1 conformational changes.

For each construct, the relative fluorescence intensities of the individual fluorophores in the various VSD states are shown based on the fit parameters, taking the intensity of the resting state as 100%. Values of different constructs cannot be directly compared to each other.
