# Supplementary material for: Multiple mechanisms contribute to fluorometry signals from the voltage-gated proton channel
Source: Commun Biol. 2022 Oct 26;5:1131. doi: 10.1038/s42003-022-04065-6 (PMC9606259; doi:10.1038/s42003-022-04065-6)
Supplement: Supplementary file 3 — Reporting Summary [file 42003_2022_4065_MOESM3_ESM.pdf]

nature portfolio

Corresponding author(s): Ferenc Papp

Last updated by author(s): Sep 9, 2021

Reporting Summary

Nature Portfolio wishes to improve the reproducibility of the work that we publish. This form provides structure for consistency and transparency in reporting. For further information on Nature Portfolio policies, see our Editorial Policies and the Editorial Policy Checklist.

Statistics

For all statistical analyses, confirm that the following items are present in the figure legend, table legend, main text, or Methods section.

n/a	Confirmed	
☐	☒	The exact sample size (n) for each experimental group/condition, given as a discrete number and unit of measurement
☐	☒	A statement on whether measurements were taken from distinct samples or whether the same sample was measured repeatedly
☐	☒	The statistical test(s) used AND whether they are one- or two-sided<br>*Only common tests should be described solely by name; describe more complex techniques in the Methods section.*
☐	☒	A description of all covariates tested
☐	☒	A description of any assumptions or corrections, such as tests of normality and adjustment for multiple comparisons
☐	☒	A full description of the statistical parameters including central tendency (e.g. means) or other basic estimates (e.g. regression coefficient) AND variation (e.g. standard deviation) or associated estimates of uncertainty (e.g. confidence intervals)
☐	☒	For null hypothesis testing, the test statistic (e.g. F, t, r) with confidence intervals, effect sizes, degrees of freedom and P value noted<br>*Give P values as exact values whenever suitable.*
☒	☐	For Bayesian analysis, information on the choice of priors and Markov chain Monte Carlo settings
☒	☐	For hierarchical and complex designs, identification of the appropriate level for tests and full reporting of outcomes
☒	☐	Estimates of effect sizes (e.g. Cohen's d, Pearson's r), indicating how they were calculated

Our web collection on statistics for biologists contains articles on many of the points above.

Software and code

Policy information about availability of computer code

Data collection	pClamp10 (Molecular Devices, San Jose, CA)
Data analysis	Clampfit (v10; Molecular Devices), SigmaPlot (v10; SigmaStat Software, San Jose, CA); Excel (Microsoft, Redmond, WA)

For manuscripts utilizing custom algorithms or software that are central to the research but not yet described in published literature, software must be made available to editors and reviewers. We strongly encourage code deposition in a community repository (e.g. GitHub). See the Nature Portfolio guidelines for submitting code & software for further information.

Data

Policy information about availability of data

All manuscripts must include a data availability statement. This statement should provide the following information, where applicable:

- Accession codes, unique identifiers, or web links for publicly available datasets
- A description of any restrictions on data availability
- For clinical datasets or third party data, please ensure that the statement adheres to our policy

The datasets generated and analyzed during the current study are available from the corresponding author on reasonable request.

Field-specific reporting

Please select the one below that is the best fit for your research. If you are not sure, read the appropriate sections before making your selection.

☒ Life sciences ☐ Behavioural & social sciences ☐ Ecological, evolutionary & environmental sciences

For a reference copy of the document with all sections, see nature.com/documents/nr-reporting-summary-flat.pdf

Life sciences study design

All studies must disclose on these points even when the disclosure is negative.

Sample size	Sample sizes were not predetermined. VCF measurements were carried out on individual oocytes. For a given set of experimental conditions we always recorded a minimum of 3 cells. If variability in the data seemed high (CV>20%) we performed more experiments until results were more consistent. Due to the time-consuming nature of electrophysiology experiments the published results are typically based on sample sizes lower than for many other experimental techniques. We believe that our sample sizes match or exceed the ones generally accepted for such publications.
Data exclusions	For VCF the quality of the recording is typically obvious during the experiment itself, since large leak current (typically >10% of the active current) or poor voltage-clamping are visible in the acquisition software or on the instrument panel. Such recordings are discarded immediately, without being analyzed. During analysis further recordings may be discarded if multiple parameters imply that the errors may have been introduced. For example, simultaneous presence of non-negligible leak current, abnormal activation or tail current kinetics, inappropriate response to control solution exchanges (e.g. no shift in voltage-dependent activation when there should be) can justify the exclusion of the recording. Data analysis was performed according to predetermined protocols used by our group for many years.
Replication	Each VCF experiment is performed on individual oocytes, so reproducibility can be verified from cell to cell. Due to the potential variability among oocyte batches from different frogs, each dataset includes experiments from at least 2 days meaning different batches of cells. In order to ensure that observed effects were due to the treatment and not to fluid exchange flow artifacts, in addition to the solution containing the active component we also applied control solution to the recorded cells to check for any changes in current or fluorescence.
Randomization	Experiments were performed in sequence, as the project logic required it, therefore experiments were performed on cells available on that particular day. In this respect, samples were allocated to different groups randomly. We took care to perform the same type of experiment on at least two batches of cells.
Blinding	We have not applied blinding as most of our channel constructs produce VCF signals of unique shapes and magnitudes and so are easily identified by the experimenter during the recording and data analysis.

Reporting for specific materials, systems and methods

We require information from authors about some types of materials, experimental systems and methods used in many studies. Here, indicate whether each material, system or method listed is relevant to your study. If you are not sure if a list item applies to your research, read the appropriate section before selecting a response.

Materials & experimental systems

n/a	Involved in the study	
☒	☐	Antibodies
☐	☒	Eukaryotic cell lines
☒	☐	Palaeontology and archaeology
☒	☐	Animals and other organisms
☒	☐	Human research participants
☒	☐	Clinical data
☒	☐	Dual use research of concern

Methods

n/a	Involved in the study	
☒	☐	ChIP-seq
☒	☐	Flow cytometry
☒	☐	MRI-based neuroimaging

Eukaryotic cell lines

Policy information about cell lines

Cell line source(s)	Xenopus laevis oocytes for voltage-clamp fluorometry experiments were purchased from EcoCyte Bioscience (Dortmund, Germany).
Authentication	Xenopus laevis oocytes were not authenticated.
Mycoplasma contamination	Xenopus laevis oocytes were not tested for mycoplasma contamination.

Commonly misidentified lines
(See ICLAC register)

Name any commonly misidentified cell lines used in the study and provide a rationale for their use.
